# Expression analysis of tissue factor pathway inhibitors TFPI-1 and TFPI-2 in *Paralichthys olivaceus* and antibacterial and anticancer activity of derived peptides

**DOI:** 10.1186/s13567-021-00908-y

**Published:** 2021-02-25

**Authors:** Guanghua Wang, Bing Xie, Yanli Su, Qinqin Gu, Dongfang Hao, Hongmei Liu, Changbiao Wang, Yonghua Hu, Min Zhang

**Affiliations:** 1 Institute of Tropical Bioscience and Biotechnology, Hainan Academy of Tropical Agricultural Resource, CATAS, Haikou, 571101 China; 2 Marine Science and Engineering College, Qingdao Agricultural University, Qingdao, 266109 China; 3 Laboratory for Marine Biology and Biotechnology, Pilot National Laboratory for Marine Science and Technology, Qingdao, China; 4 Hainan Provincial Key Laboratory for Functional Components Research and Utilization of Marine Bio-Resources, Haikou, 571101 China

**Keywords:** *Paralichthys olivaceus*, TFPI, C-terminal peptides, Antibacterial activity, Anticancer cell activity

## Abstract

Tissue factor pathway inhibitors (TFPI), including TFPI-1 and TFPI-2, are Kunitz-type serine protease inhibitors that mainly inhibit the blood coagulation induced by tissue factors. Previous reports on teleost proved TFPI play important roles in innate immunity. In this study, two TFPI (PoTFPI-1 and PoTFPI-2) molecules from Japanese flounder (*Paralichthys olivaceus*) were analyzed and characterized for their expression patterns, antibacterial and anticancer activities of the C-terminal derived peptides. Quantitative real time RT-PCR analysis shows that constitutive *PoTFPI-1* expression occurred, in increasing order, in the brain, muscle, spleen, gills, head kidney, blood, intestine, heart, and liver; *PoTFPI-2* was expressed, in increasing order, in the brain, gills, head kidney, muscle, intestine, spleen, liver, heart, and blood. Under the stimulation of fish pathogens, both *PoTFPI-1* and *PoTFPI-2* expressions increased significantly in a manner that depended on the pathogens, tissue type, and infection stage. Furthermore, C-terminal peptides TP25 and TP26, derived from PoTFPI-1 and PoTFPI-2, respectively, were synthesized and proved to be active against *Micrococcus luteus* (for TP25 and TP26) and *Staphylococcus aureus* (for TP25) via retardation effects on bacterial nucleic acids. In addition, TP25 and TP26 also displayed significant inhibitory effects on human colon cancer cell line HT-29. These results reveal that both PoTFPI-1 and PoTFPI-2 play important roles in host innate immunity. The antibacterial activity and anticancer cells function of TP25 and TP26 will add new insights into the roles of teleost TFPI.

## Introduction

Tissue Factor (TF, thromboplastin), as the major factor for the initiation of the blood coagulation process, is a high-affinity receptor for the coagulation factor VII (FXII). It initiates the extrinsic pathway of blood coagulation by binding in the presence of calcium to FXII, and formats TF–FVII/FVIIa complex, which activates FX and FIX factors, leading to the generation of thrombin and the conversion of blood fibrinogen to fibrin [1–3]. Tissue Factor Pathway Inhibitor (TFPI), a Kunitz-type serine protease inhibitor, is the natural direct inhibitor of the TF/FVIIa complex, and the formation of the inhibitory complex blocks the activation of the coagulation cascade [4]. Currently, two members in the TFPI family, TFPI-1 and its structural homologue TFPI-2, are found in mammals [5, 6]. Structurally, there are three bovine pancreatic trypsin inhibitors (BPTI)/Kunitz domains in TFPI-1. Different BPTI/Kunitz domains have different functions. The first domain mainly binds FVIIa and the second domain binds FXa. However, the third domain is involved in cell surface and lipoprotein association [7, 8]. By means of alternative RNA splicing, TFPI-1 emerges two isoforms, TFPIα and TFPIβ [9]. There are a K3 domain and basic C-terminal region in TFPIα [9], while TFPIβ contains a region involved in the attachment of a GPI membrane anchor [10]. TFPI-2 is a structural analogue of TFPI, also a potent endogenous inhibitor of tissue factor (TF)-mediated coagulation [11]. Human TFPI-2 is synthesized and secreted by multiple cells, such as smooth muscle cells, skin fibroblasts, and endothelial cells [12–14]. TFPI-2 has many functions. It is reported that human TFPI-2 is involved in homeostasis in umbilical vein endothelial cells and trophoblast cells [5, 15]. And TFPI-2 also plays roles in the regulation of extracellular matrix remodeling and participates in tumor growth and metastasis [16–18]. Whether it is TFPI-1 or TFPI-2, their C-terminal regions can be cleaved off at different positions, resulting in TFPI truncates with various sizes [19].

It has been found that human TFPI exhibit serine protease inhibitor activity against plasmin, trypsin, chymotrypsin, plasma kallikrein, factor VIIa, and cathepsin G [5, 13, 20–22]. In addition to functioning as a major regulator of blood coagulation, mammalian TFPI is involved in immunity response. It is reported that human TFPI-1 is able to reduce mortality in rabbit models for septic shock induced by lethal bacterial challenge [23]. Expression of TFPI-1 and TFPI-2 are increased under stimulation with inflammatory mediators, endotoxin, or molecules involved in infection and inflammation [24, 25]. In contrast to mammalian TFPI, which have been studied extensively in recent years, fish TFPI is not well developed. To date, TFPI-1 and/or TFPI-2 have only been discovered in several fish species, including zebrafish (*Danio rerio*), common carp (*Cyprinus carpio*), red drum (*Sciaenops ocellatus*), and half-smooth tongue sole (*Cynoglossus semilaevis*) [26–29].

Japanese flounder (*Paralichthys olivaceus*) is a flat fish cultured widely in China as an economic species. Currently, the fish has suffered serious diseases, and the studies of its responses to pathogen infection are limited. In this study, the sequence signatures of Japanese flounder TFPI-1 and TFPI-2 (named as PoTFPI-1 and PoTFPI-2, respectively) were analyzed, and their expression patterns under normal or pathogen stimulation conditions were investigated. Moreover, the antibacterial and anticancer cell activity of the C-terminal peptides of PoTFPI-1 and PoTFPI-2 (named as TP25 and TP26) were examined.

## Materials and methods

### Fish

Healthy flounder (average 19.2 ± 1.5 g) were purchased from a commercial fish farm in the Shandong Province, China, and maintained at 20 ℃ in aerated seawater. Fish were acclimatized in the laboratory for two weeks before experimental manipulation. Before experimental manipulation, fish were randomly sampled and verified to be absent of pathogens in tissues as reported previously [30, 31]. Before tissue collection, fish were euthanized with an overdose of tricaine methanesulfonate (Sigma, St. Louis, MO, USA) as reported previously [28].

### Bacterial and viral strains

The pathogens *Edwardaiella tarda*, *Klebsiellar pneumonia*, *Pseudomonas putida*, *Serratia marcescens*, *Streptococcus agalactiae*, *Vibrio anguillarum*, *Vibrio harveyi*, *Vibrio litoralis*, *Vibrio parahaemolyticus* and *Vibrio scophthalmi* have been previously preserved in the laboratory. *Escherichia coli* DH5α was purchased from Tiangen (Beijing, China), *Micrococcus luteus* 1D00051, *Staphylococcus aureus* 1D00101 and *Vibrio vulnificus* 1H00066 were purchased from China General Microbiological Culture Collection Center (Beijing, China). Except for *S. agalactiae*, which was cultured in Brain Heart Infusion (BHI) broth, all other strains were cultured in Luria–Bertani (LB) medium. All strains were cultured at 37 °C (for *E. coli* and *M. luteus*) or 28 °C (for all others). ISKNV was propagated in a continuous cell line (named as CPB) established previously from the brain of *Siniperca chuatsi* [32]*.*

### Sequence analysis of TFPI-1 and TFPI-2

The cDNA sequences of PoTFPI-1 and PoTFPI-2 are available from GenBank database (GenBank accession numbers XP_019955331 and XM_020090927). The cDNA and amino acid sequences of TFPI-1 and TFPI-2 were analyzed using the BLAST program at the National Center for Biotechnology Information (NCBI). The molecular mass and theoretical isoelectric point (pI) were analyzed with the ExPASy server. The secondary structure of TFPI could be browsed on the Pôle Bioinformatique Lyonnais (PBIL) server. The cleavage site for the signal peptide and protein domains was analyzed using the SMART program. The presumed 3D protein structural model was established using protein homology/analogy recognition engine V 2.0 (Phyre2). The phylogenetic tree was constructed based on the amino acid sequences of TFPI from various species retrieved from GenBank, with the MEGA 6.0 software package. Data were analyzed using Poisson correction, and gaps were removed by pairwise deletion. The reliability of the tree was assessed by 1000 bootstrap repetitions.

### Quantitative real time polymerase chain reaction (RT-qPCR) analysis of PoTFPI-1 and PoTFPI-2 expression in fish tissues under normal physiological conditions

Brain, heart, gills, head kidney, spleen, liver, muscle, blood and intestines were taken aseptically from five flounder and used for total RNA extraction with the RNAprep Tissue Kit (Tiangen, Beijing, China). One microgram of total RNA was used for cDNA synthesis with the Superscript II reverse transcriptase (Invitrogen, Carlsbad, CA, USA). RT-qPCR was carried out in a LightCycler 96 system (Roche Applied Science, North Carolina, USA) using the SYBR ExScript RT-qPCR Kit (Takara, Dalian, China) as described previously [27]. The flounder β-actin gene was used as an internal control. The primers were listed in Table 1. Melting curve analysis was carried out at the end of each PCR to confirm the specificity of PCR products. The expressions of TFPI-1 and TFPI-2 were analyzed using comparative threshold cycle method (2^−ΔΔCT^). All data are given in terms of relative mRNA levels to that of tissues in which TFPI-1 or TFPI-2 expression was the lowest.

**Table 1:** Primers used in quantitative real-time PCR

Primers	Sequences (5′-3′)
Po-Actin RTF	ACCGCTGCCTCCTCCTCAT
Po-Actin RTR	TCGGGACAACGGAACCTCTC
TFPI-1 RTF	GATGTTGTCCAAGCAACTGAAG
TFPI-1 RTR	GACTGAAGCACAGCCTCTTAT
TFPI-2 RTF	GGAAATGCTCGGCCTCTATTA
TFPI-2 RTR	CTCTGCCTGGAGACAAAGTT

### 2RT-qPCR analysis of PoTFPI-1 and PoTFPI-2 expressions upon bacterial and viral infection

Infections with various pathogens were performed as reported previously [33]. Briefly, *V. anguillarum* and *E. tarda* were cultured as above to an OD_600_ of 0.8. The cells were washed and resuspended in PBS to 10^6^ CFU/mL. ISKNV was resuspended in PBS to 1 × 10^7^ copies/mL. Fish were divided randomly into four groups (30 individuals/group) and injected intraperitoneally (i.p.) with 100 µL *V. anguillarum, E. tarda,* ISKNV or PBS per fish, and maintained at 20 ℃. Five fish were euthanized at 0 h, 6 h, 12 h, 24 h and 48 h, or 0 day, 1 day, 3 days, 5 days and 7 days (for ISKNV infection group) post-infection. Considering that head kidney, liver, and spleen were the main immune organs in fish and were easy to be obtained, so the three tissues were collected under aseptic condition. Total RNA extraction, cDNA synthesis, and RT-qPCR were performed as described above. The primers used are listed in Table 1.

### Peptide synthesis

FITC-labeled and unlabeled TP25 (RKQCIRKCIRRREPHGKMMIRIRRK) of PoTFPI-1, corresponding to the C-terminal residues 253 to 277, TP26 (GEKKYRSQRKIRRMRRKRKYPSFMQA) of PoTFPI-2, corresponding to the C-terminal residues 200 to 225, and the control peptide P86P15 [33] were synthesized by Pepmic (Suzhou, China). The peptides were purified by high-performance liquid chromatography to 95% purity. Lyophilized peptides were stored at −20 °C and dissolved in PBS (pH 6.5) before use.

TP25 of PoTFPI-1, corresponding to the C-terminal residues 253 to 277, has a pI of 12.01 and contains thirteen strongly basic amino acids, six hydrophobic amino acids, and six polar amino acids. TP26 of PoTFPI-2, corresponding to the C-terminal residues 200 to 225, has a pI of 11.90 and contains twelve strongly basic amino acids, five hydrophobic amino acids, and nine polar amino acids.

### Antibacterial spectrum

To carry out antibacterial spectrum assay, bacteria mentioned above were cultured to mid-logarithmic phase. Then the cells were centrifuged, washed, and resuspended in PBS to 2 × 10^6^ CFU/mL. Fifty microliters of the suspension were plated on LB agar plates, the sterile filter papers were slipped onto the LB plates, and 5 μL of each peptide was added to the filter paper. All plates were cultured as above for 24 h, and the anti-bacterial effect was determined according to the presence of an inhibition zone. The assay was performed three times.

### Antibacterial activity

Antibacterial activities of TP25 and TP26 were evaluated using minimum inhibitory concentration (MIC) and minimum bactericidal concentration (MBC) assays. The target bacteria screened by spectrum assay were cultured as described above to mid-logarithmic phase. The bacteria were centrifuged, washed, and resuspended in PBS to 2 × 10^6^ CFU/mL. TP25 and TP26 were dissolved in PBS and made two-fold serial dilutions. The dilution was mixed with the bacterial suspension in 96-well polypropylene microtiter plates and incubated for 24 h. Peptide P86P15 was used as a negative control. The MIC was then calculated as the lowest peptide concentration that yielded no visible growth. The culture was plated on LB agar plates and incubated for 48 h. Then the colonies growing on the plates were counted. MBC was defined as the lowest peptide concentration that resulted in no colony emergence on the plates. The assay was performed three times.

### Cell location of TP25 and TP26

Cell locations of TP25 and TP26 were examined as reported previously [34]. Briefly, *M. luteus* was cultured as above and resuspended in PBS to 2 × 10^6^ CFU/mL. FITC-labeled TP25, TP26, or P86P15 were incubated with 20 μL bacterial cells at room temperature for 0.5 h. The cells were washed with PBS, then 0.4% trypan blue was added into the cells and incubated at room temperature for 0.5 h to quench the extracellular fluorescence. After washing with PBS, the cells were observed with a fluorescence microscope (Leica DM1700, Germany).

### Effect of TP25 and TP26 on DNA

To evaluate the effect of TP25 and TP26 on bacterial genomic DNA, genomic DNA of *M. luteus* was extracted with TIANamp Bacteria DNA Kit (Tiangen, Beijing, China). One hundred nanograms genomic DNA of *M. luteus* was mixed with different concentrations of TP25, TP26, P86P15, or PBS, in a total volume of 10 μL, respectively. The mixture was incubated at room temperature for 30 min and subjected to agarose gel electrophoresis. For mixtures in which the genomic DNA disappeared in the agarose gel electrophoresis, two μL of proteinase K was added into the tube and incubated for 10 min, then the reaction products were analyzed by agarose gel electrophoresis.

### Effect of TP25 and TP26 on RNA

To check the effect of TP25 and TP26 on bacterial RNA, total RNA of *M. luteus* were extracted with RNAprep Pure Cell/Bacteria Kit (Tiangen, Beijing, China). One hundred nanograms RNA of *M. luteus* were mixed with different concentrations of TP25, TP26, P86P15, or PBS in a total volume of 10 μL. The mixture was incubated at room temperature for 30 min and subjected to agarose gel electrophoresis.

### Inhibitory effect of TP25 and TP26 on human cancer cells HT-29

Human colon cancer cell line HT-29 (iCell-h078) was cultured in McCOY 5A medium (iCell-0011) supplemented with 10% fetal bovine serum, 100 Units/mL penicillin, and 100 μg/mL streptomycin in a water-saturated atmosphere containing 5% CO_2_ at 37 °C. Cells were detached with 2 mL trypsin/EDTA (iCell Bioscience Inc) for 2 min at 37 °C, 3 mL of complete medium was added and the cells were collected by low-speed centrifugation. The cell pellet was suspended in culture medium, and 100 μL of a cell suspension (5 × 10^4^ cells/ml) were added into a 96 well tissue culture plate. After incubation for 24 h at 37 °C, the cells were washed twice with PBS and the medium was replaced with fresh McCOY’s 5A medium without fetal bovine serum but containing P86P15, TP25 or TP26 at concentrations of 50, 300, and 500 μM, respectively. Then the cells were incubated for 24 h at 37 °C. After incubation with peptides, the cells were dyed by trypan blue and observed for morphological changes under an inverted phase contrast microscope (Zeiss, Germany). At the same time, the survival rate of HT-29 cells was evaluated by MTT assay. Briefly, 15 mg of 2, 3-bis [2-methyloxy-4-nitro-5-sulfophenyl]-2H-tetrazolium-5-carboxanilide (MTT, Solarbio) was completely dissolved in 3 ml PBS and sterile filtered. The above cell culture medium was replaced with 30 μL MTT solution. After incubation for 4 h at 37 °C, the absorbance at 540 nm was recorded using a microplate reader. Experiments were executed in triplicate. The results are presented as a percentage of the inhibition rate for viable cells.

### Statistical analysis

All statistical analyses were performed with SPSS 25.0 software (SPSS Inc., Chicago, IL, USA). Data were analyzed with analysis of variance (ANOVA), and statistical significance was defined as *P* < 0.05.

## Results

### Sequence analysis and structure characteristics of PoTFPI-1 and PoTFPI-2

**Figure 1:**
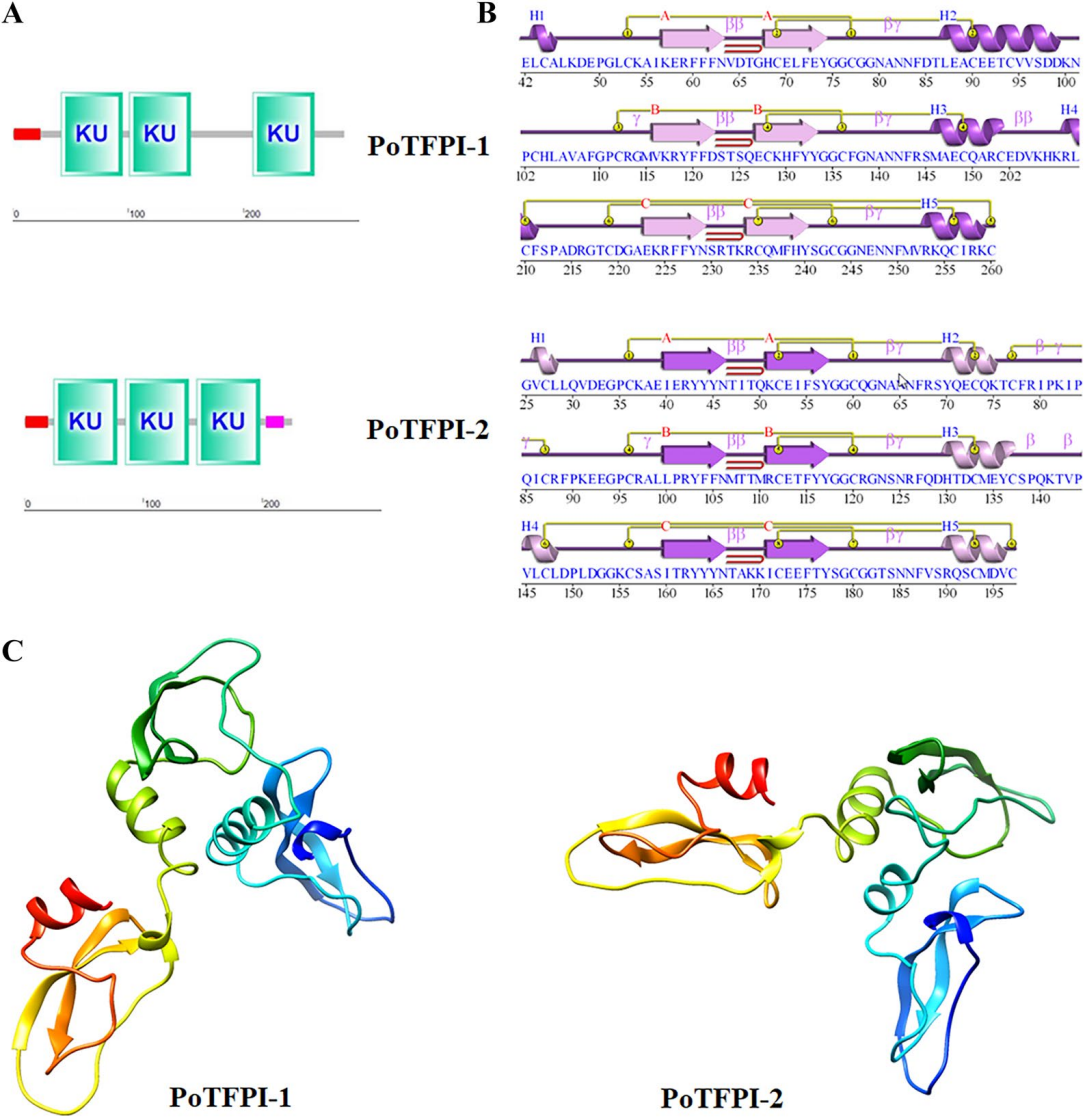
**Structural domains and spatial structures of PoTFPI-1 and PoTFPI-2.** **A**, the predicted structural domain of protein motifs of PoTFPI-1 and PoTFPI-2. The signal peptide is indicated with a red rectangle. The KU domains is indicated with green rectangles. The region of low compositional complexity is indicated with a pink rectangle. **B** the secondary structures of protein motifs of PoTFPI-1 and PoTFPI-2. The spatial structures are predicted by PDBsum Generate. **C** the spatial structures of PoTFPI-1 and PoTFPI-2. The 3D structure of TFPI-1 predicted using Phyre2. Model based on template c4bd9B, 164 residues (57% of the TFPI-1) have been modelled with 100.0% confidence by the single highest scoring template, and the image coloured by rainbow N(bule) → C(red)terminus. Model dimensions (Å): X:39.349 Y:67.655 Z:57.454. The 3D structure of TFPI-2 predicted using Phyre2. Model based on template c4bd9B, 164 residues (73% of TFPI-2) have been modelled with 100.0% confidence by the single highest scoring template, and the image coloured by rainbow N(bule) → C(red)terminus. Model dimensions (Å): X:37.322 Y:66.594 Z:54.944.

The cDNA sequence of the gene PoTFPI-1 contains 864 bp ORF which codes for 287 amino acid residues with a calculated molecular mass of 32.55 kDa and a theoretical pI of 8.41. PoTFPI-1 contains a putative signal peptide sequence (residues 1–24) and three Kunitz (KU) domains (residues 42–95, 101–154, and 208–261, respectively) (Figure 1A). The cDNA sequence of the gene PoTFPI-2 contains 678 bp ORF which codes 225 amino acid residues with a calculated molecular mass of 26.11 kDa and a theoretical pI of 8.84. PoTFPI-2 contains a putative signal peptide sequence (residues 1–20), three KU domains (residues 25–78, 85–138, and 145–198, respectively), and a region of low compositional complexity (LCR, residues 203–218) (Figure 1A).

There are three sheets, three beta hairpins, three beta bulges, six strands, five helices, two helix-helix interactions, eleven beta turns, four gamma turns and seven disulphides in the spatial structure of PoTFPI-1. The three KU domains each contain a beta hairpin (Figure 1B). There are three sheets, three beta hairpins, three beta bulges, six strands, five helices, two helix-helix interactions, twelve beta turns, six gamma turns and eight disulphides in the spatial structure of PoTFPI-2. With a similar structure to PoTFPI-1, the three KU domains in PoTFPI-2 each contain a beta hairpin (Figure 1B). Meanwhile, the 3D structures of PoTFPI-1 and PoTFPI-2 were predicted by Phyre2 with 100% confidence, and the image was colored by rainbow from N to C terminus. The results show that the PoTFPI-1 protein is categorized as an alpha helix (11%) and beta turn (16%) (Figure 1C). PoTFPI-2 protein is categorized as an alpha helix (22%) and beta turn (18%) (Figure 1C). The prediction of the tertiary structure basically conforms to the secondary structure.

**Figure 2:**
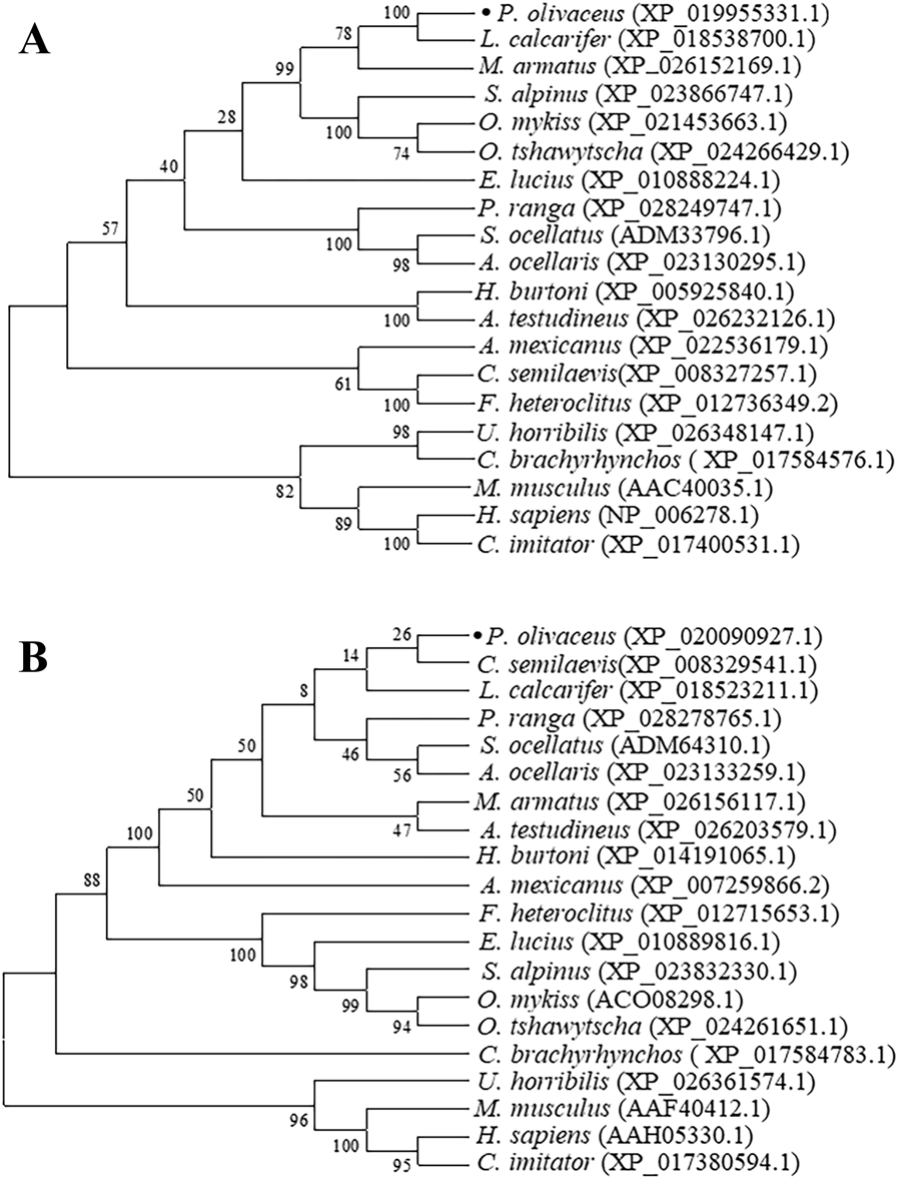
**Phylogenetic analysis of PoTFPI-1 (A) and PoTFPI-2 (B) homologues.** Phylogenetic trees were constructed based on the amino acid sequences of TFPI-1 or TFPI-2 proteins from different organisms. The numbers at the forks indicate the bootstrap values. The dark solid circle represents *Paralichthys olivaceus* TFPI-1. The species and the GenBank accession numbers are as follows: *Paralichthys olivaceus* (XP_019955331.1), *Homo sapiens* (NP_006278.1), *Mus musculus* (AAC40035.1), *Sciaenops ocellatus* (ADM33796.1), *Lates calcarifer* (XP_018538700.1), *Amphiprion ocellaris* (XP_023130295.1), *Cynoglossus semilaevis*(XP_008327257.1), *Mastacembelus armatus* (XP_026152169.1), *Parambassis ranga* (XP_028249747.1), *Astyanax mexicanus* (XP_022536179.1), *Oncorhynchus mykiss* (XP_021453663.1), *Esox lucius* (XP_010888224.1), *Oncorhynchus tshawytscha* (XP_024266429.1), *Salvelinus alpinus* (XP_023866747.1), *Fundulus heteroclitus* (XP_012736349.2), *Haplochromis burtoni* (XP_005925840.1), *Anabas testudineus* (XP_026232126.1), *Ursusarctos horribilis* (XP_026348147.1), *Cebuscapucinus imitator* (XP_017400531.1), *Corvus brachyrhynchos* ( XP_017584576.1).

To reveal the molecular phylogenetic positions of PoTFPI-1 and PoTFPI-2, phylogenetic trees were constructed based on deduced amino acid sequences from teleosts, mammals, and aves. The results show that PoTFPI-1 was clustered most closely with the TFPI-1 protein of *L. calcarifer* and PoTFPI-2 was clustered most closely with the TFPI-2 proteins of *C. semilaevi*, which reveals phylogenetic positions of PoTFPI-1 and PoTFPI-2, and their strong relationship with their counterparts in other species (Figure 2).

### Expressions of PoTFPI-1 and PoTFPI-2 under normal physiological conditions

**Figure 3:**
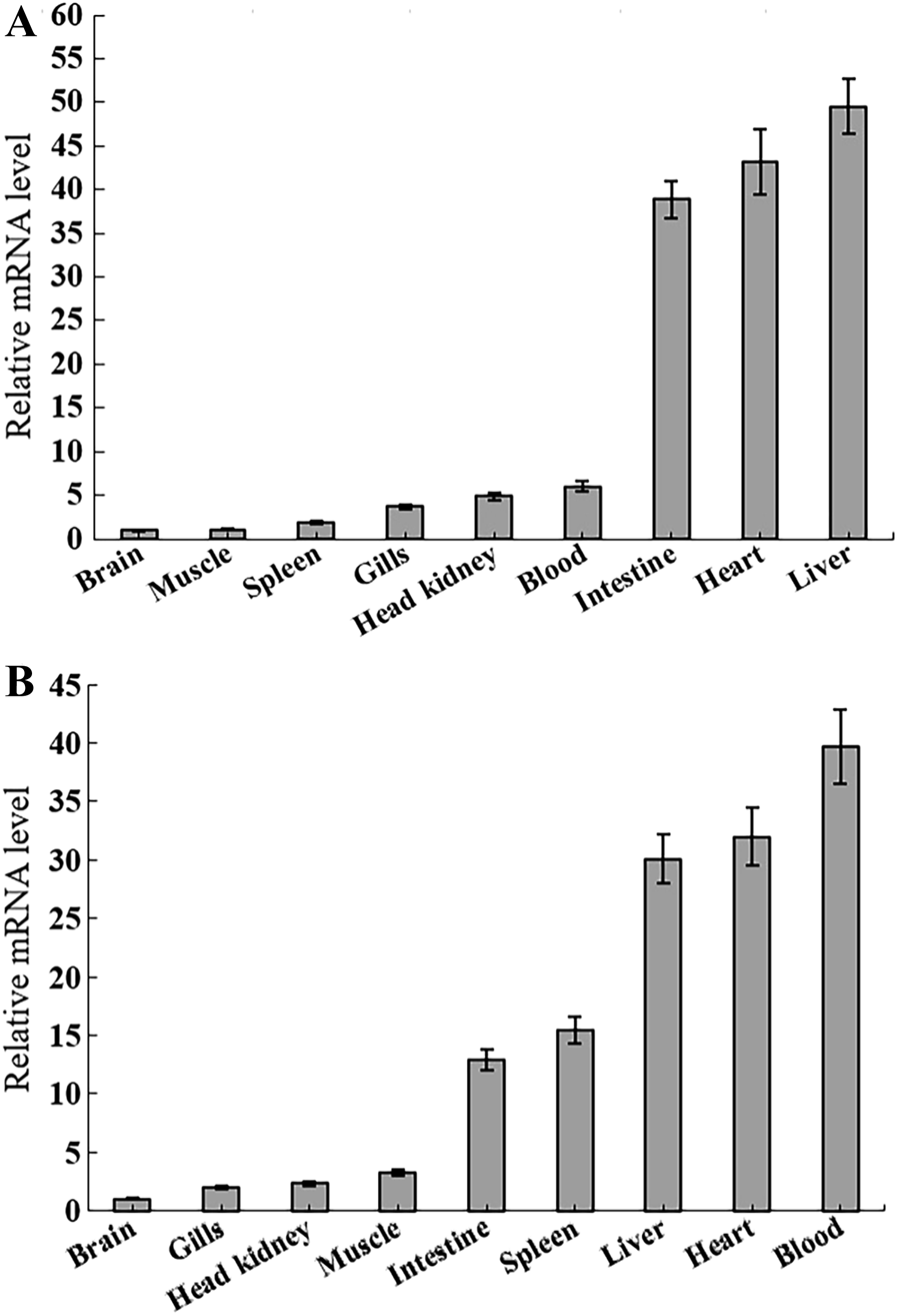
***PoTFPI-1*** **and** ***PoTFPI-2*** **expression in flounder tissues.** *PoTFPI-1* and *PoTFPI-2* expressions in the brain, muscle, spleen, gill, head kidney, blood, intestine, heart, and liver of flounder were determined by quantitative real time RT-PCR. For convenience of comparison, the expression level in the brain was set as 1. Data are the means of three independent assays and presented as means ± SEM (*N* = 3). N represents the number of times the experiment was performed.

RT-qPCR was carried out to examine the expression profiles of *PoTFPI-1* and *PoTFPI-2* in different tissues of flounder under normal physiological conditions. The results show that *PoTFPI-1* and *PoTFPI-2* expression was distributed in all the examined tissues. *PoTFPI-1* expression was detected, in increasing order, in the brain, muscle, spleen, gill, head kidney, blood, intestine, heart, and liver (Figure 3A). *PoTFPI-2* expression was detected, in increasing order, in the brain, gills, head kidney, muscle, intestine, spleen, liver, heart, and blood. The expression of *PoTFPI-1* and *PoTFPI-2* in the blood is significantly higher than that in the brain (Figure 3B).

### Expression profiles of PoTFPI-1 and PoTFPI-2 upon experimental infection with bacterial and viral pathogens

**Figure 4:**
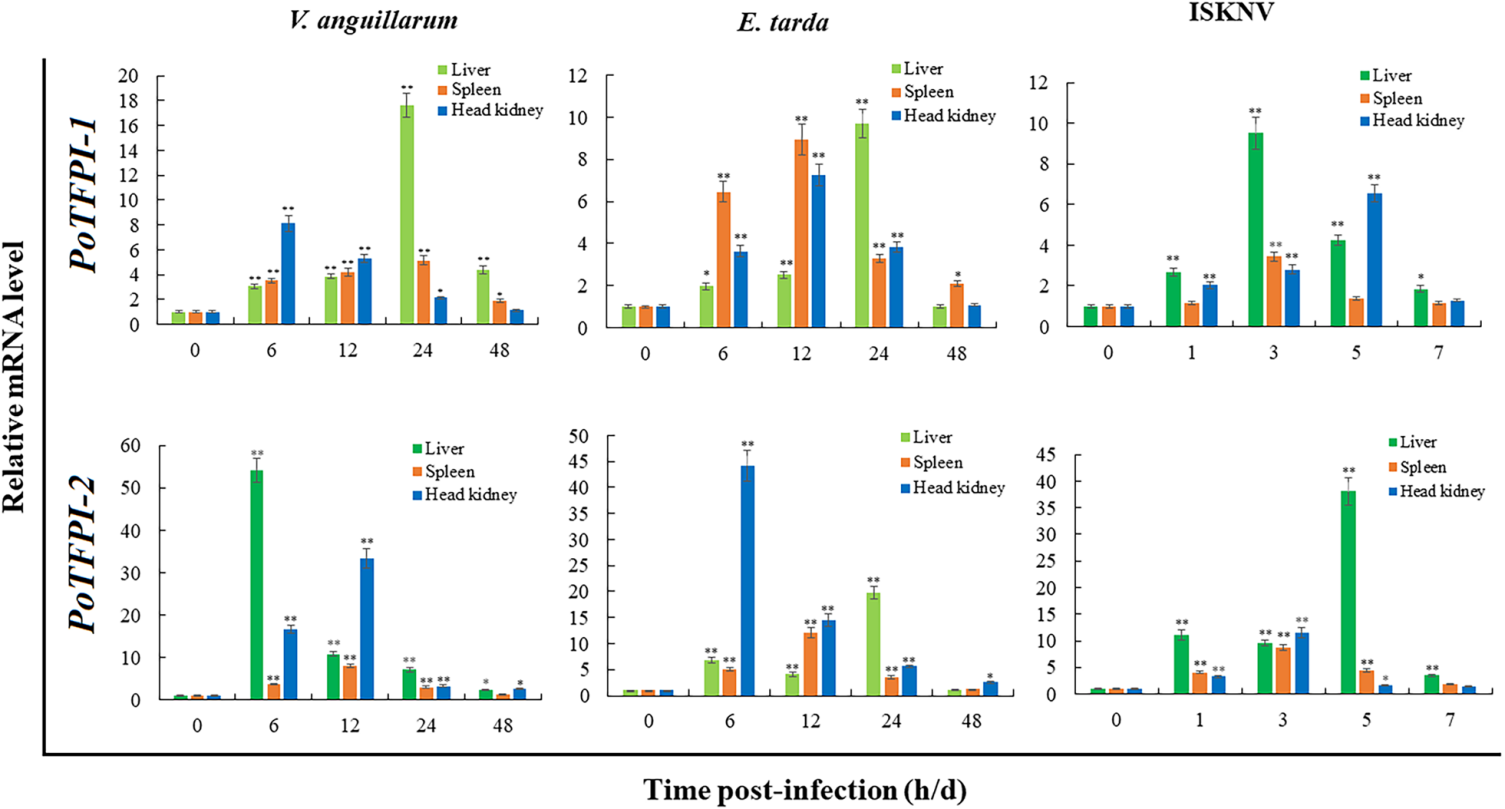
***PoTFPI-1*** **and** ***PoTFPI-2*** **expressions in response to pathogen challenge.** Japanese flounder were infected with the extracellular bacterial pathogen *Vibrio anguillarum*, the intracellular bacterial pathogen *Edwardsiella tarda*, the viral pathogen fish infectious spleen and kidney necrosis virus (ISKNV), or PBS (as the control). After infection, head kidney, spleen, and liver were taken aseptically at 6, 12, 24, and 48 h post-infection (hpi) for bacterial infection, and at 0, 1, 3, 5, and 7 days post-infection (dpi) for viral infection. The *PoTFPI-1* and *PoTFPI-2* expressions in three tissues were determined by RT-qPCR at various time points. In each case, the expression level at 0 h was set as 1. Values are shown as mean ± SEM (*N* = 3). N represents the number of times the experiment was performed. *P < 0.05, **P < 0.01.

To examine the expression patterns of *PoTFPI-1* and *PoTFPI-2* upon fish pathogen infection, flounder were challenged experimentally with intracellular pathogen *E. tarda*, extracellular pathogen *V. anguillarum*, and viral pathogen ISKNV. Total RNA was extracted from the tissues at different times and cDNA was synthesized. Then the expression of *PoTFPI-1* and *PoTFPI-2* in the liver, spleen, and head kidney were determined by RT-qPCR. The results show that expression patterns of *PoTFPI-1* and *PoTFPI-2* in a manner depended on the nature of the pathogen, tissue type, and infection time. Specifically, upon the infection of *V. anguillarum*, *PoTFPI-1* and *PoTFPI-2* expressions in three immune tissues were almost all significantly up-regulated at all examined time points. For *PoTFPI-1*, the maximum induction occurred 24 h post-infection (hpi) (17.6-fold), 24 hpi (5.1-fold), and 6 hpi (8.1-fold) in the liver, spleen, and head kidney, respectively. The expression of *PoTFPI-2* peaked at 6 hpi (54.2-fold), 12 hpi (8.0-fold) and 12 hpi (33.4-fold) in the liver, spleen, and head kidney, respectively (Figure 4).

When infected by *E. tarda*, *PoTFPI-1* and *PoTFPI-2* expressions in three immune tissues were significantly up-regulated at all examined time points except 48 hpi. For *PoTFPI-1*, the maximum induction occurred at 24 hpi (9.7-fold), 12 hpi (8.9-fold), and 12 hpi (7.3-fold) in the liver, spleen, and head kidney, respectively. The expression of *PoTFPI-2* peaked at 24 hpi (19.8-fold), 12 hpi (12.0-fold) and 6 hpi (44.2-fold) in the liver, spleen, and head kidney, respectively (Figure 4).

During ISKNV infection, *PoTFPI-1* expression in the liver was all significantly enhanced at all examined time points and peaked at 3 days post-infection (dpi) (9.5-fold). In the spleen, *PoTFPI-1* expression was up-regulated only at 3 dpi (3.4-fold). In the head kidney, *PoTFPI-1* expression was enhanced at 1 dpi, 3 dpi, 5 dpi and peaked at 5 dpi (6.5-fold). *PoTFPI-2* expression in the liver was all significantly enhanced at all examined time points and peaked at 5 dpi (38.0-fold). In the spleen, *PoTFPI-2* expression was up-regulated at all examined time points except 7 dpi and peaked at 3 dpi (8.7-fold). In the head kidney, *PoTFPI-2* expression was enhanced only at 1 dpi and 3 dpi, and peaked at 3 dpi (11.6-fold) (Figure 6).

In general, the expression level of *PoTFPI-2* induced by pathogens was higher than that of *PoTFPI-1*. The significantly induced expressions by different pathogen infections indicate that PoTFPI-1 and PoTFPI-2 participate in the host anti-infectious immunity.

### Antibacterial activity of TP25 and TP26

Since PoTFPI-1 and PoTFPI-2 are highly positively charged at the C-terminus, we examined whether two short peptides TP25 and TP26, based on the C-terminus residues of PoTFPI-1 and PoTFPI-2, respectively, have any antibacterial effect.

Bactericidal analysis showed that TP25 has antibacterial activity against Gram-positive bacteria *M. luteus* and *S. aureus*, but not against other bacteria examined, including *E. tarda*, *K. pneumonia*, *P. putida*, *S. marcescens*, *S. agalactiae*, *V. anguillarum*, *V. harveyi*, *V. litoralis*, *V. parahaemolyticus*, *V. scophthalmi,* and *Vibrio vulnificus*. TP26 is effective only against *M. luteus*, not other examined bacteria. The MIC and MBC of TP25 against *M. luteus* were 5 and 20 μM, respectively. The MIC and MBC of TP25 against *S. aureus* were 3 and 20 μM, respectively. The MIC and MBC of TP26 against *M. luteus* were 120 and 550 μM, respectively (Table 2). In contrast, the peptide P86P15, which is unrelated to PoTFPI-1 and PoTFPI-2, had no antibacterial effect on *M. luteus* and *S. aureus*.

**Table 2:** MIC and MBC of TP25 and TP26 against bacteria

Strains	TP25		TP26	
	MIC (μM)	MBC (μM)	MIC (μM)	MBC (μM)
*Micrococcus luteus*	5	20	120	550
*Staphylococcus aureus*	3	20		

### Localization of TP25 and TP26 in *M. luteus*

**Figure 5:**
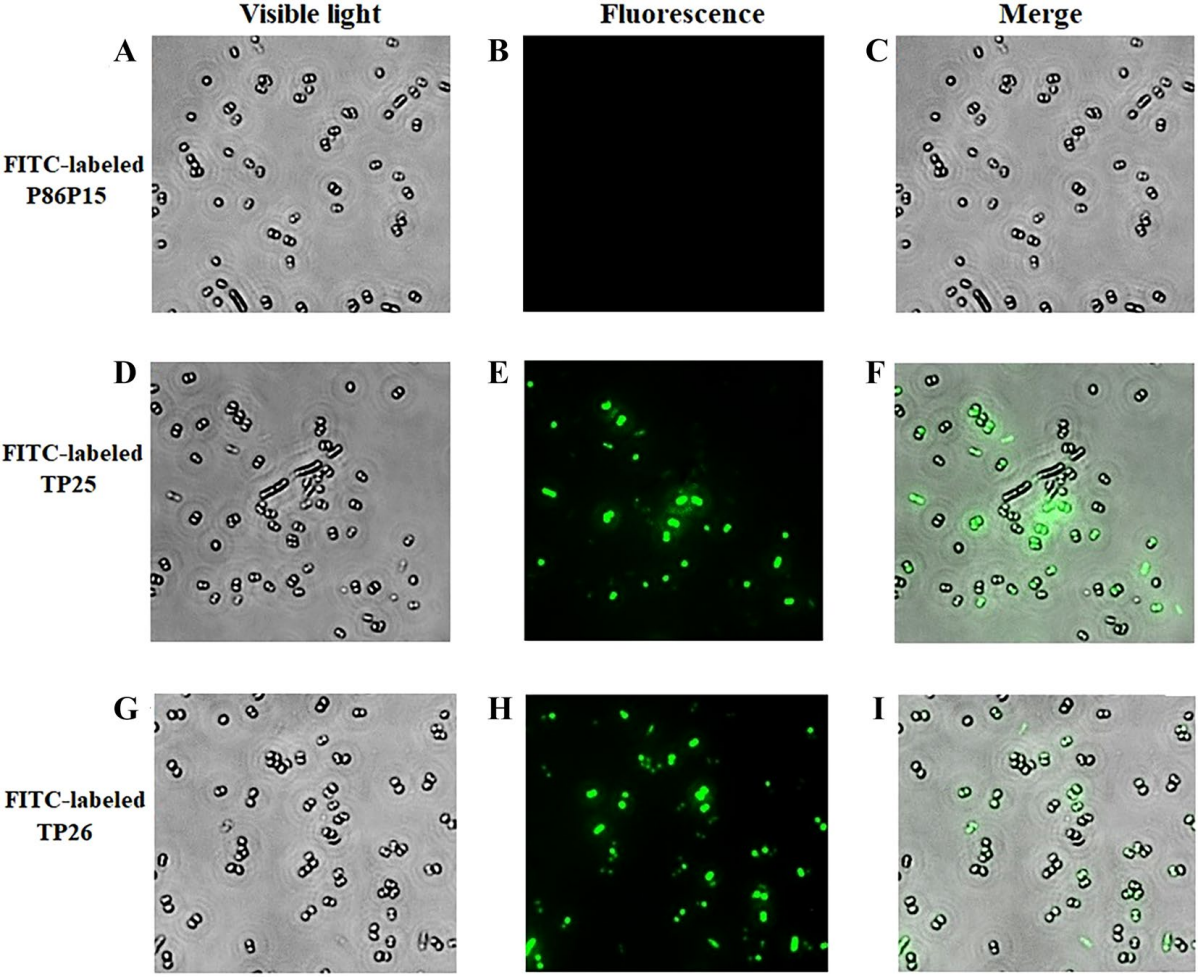
**Penetration of TP25 and TP26 into the target bacterial cells.** *Micrococcus luteus* was incubated with FITC-labeled TP25 (**D** and **E**), FITC-labeled TP26 (**G** and **H**) or FITC-labeled control peptide P86P15 (**A** and **B**) for 0.5 h. After quenching extracellular fluorescence, the cells were observed as above under a microscope with (**B**, **E** and **H**) or without (**A**, **D** and **G**) fluorescence. **C**, merged image of **A** and **B**; **F**, merged image of **D** and **E**; **I**, merged image of **G** and **H**. Magnification, 4000 × .

In order to investigate the cellular location of TP25 and TP26, FITC-labeled TP25, TP26, or P86P15 was incubated with *M. luteus*, which is the common target bacteria of TP25 and TP26. After quenching the extracellular fluorescence, intracellular accumulation of peptides was visualized using fluorescence microscopy. The results show that fluorescence was observed in most of the cells incubated with FITC-labeled TP25 and FITC-labeled TP26. However, no fluorescence was observed in the cells incubated with FITC-labeled P86P15 (Figure 5), which indicates TP25 and TP26 have the ability of penetrating into *M. luteus*.

### Effects of TP25 and TP26 on bacterial genomic DNA

**Figure 6:**
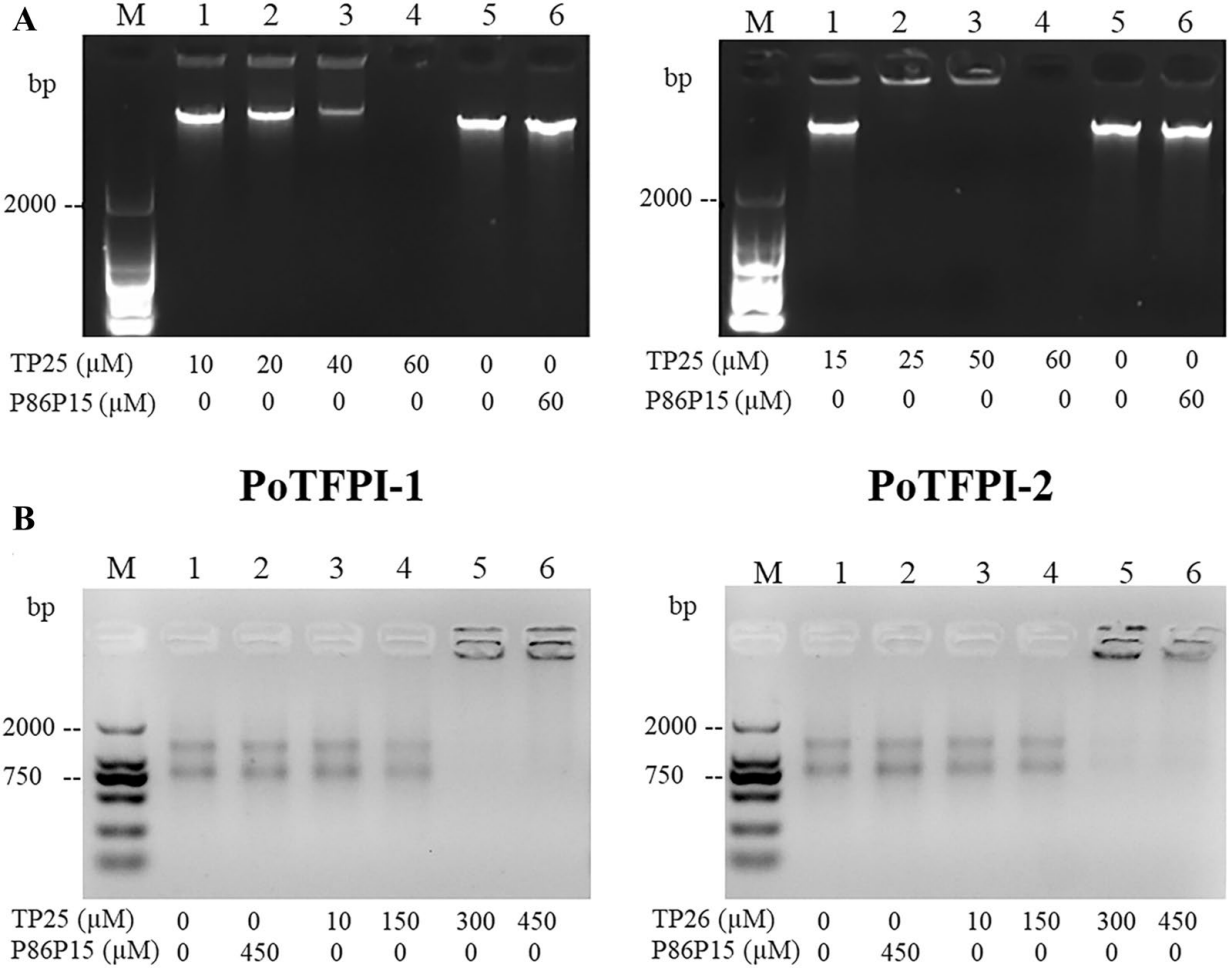
**In vitro effects of TP25 and TP26 on bacterial genomic DNA and total RNA.** **A** the genomic DNA of *Micrococcus luteus* was incubated with different concentrations of TP25, TP26, or P86P15, then the mixture was subjected to electrophoresis. **B** the total RNA of *Micrococcus luteus* was incubated with different concentrations of TP25, TP26, or P86P15, then the mixture was subjected to electrophoresis.

Since some antibacterial peptides possess the ability of interacting with genomic DNA, we wondered whether TP25 and TP26 had any effect on genomic DNA. To investigate this question, the genomic DNA of *M. luteus* was chosen based on the results of antibacterial activity of TP25 and TP26, and the in vitro effects of TP25 and TP26 on genomic DNA of *M. luteus* were analyzed. The results show that TP25 could bind with genomic DNA and caused a gel retardation effect. When the concentration of TP25 was 60 μM, the genomic DNA disappeared (Figure 6A). In order to investigate whether or not the genomic DNA was degraded, the TP25 in the mixture was degraded with Protease K, and the following electrophoretic analysis indicated that the genomic DNA was still intact (data not shown), which further indicates that TP25 could bind with genomic DNA. On the contrary, the control P86P15 had no effect on bacterial genomic DNA (Figure 6A). Similar results were observed when TP26 was incubated with genomic DNA of *M. luteus* (Figure 6A). These findings suggest that TP25 and TP26 have a direct interaction with bacterial genomic DNA.

### Effects of TP25 and TP26 on total RNA

Considering the effect exerted by TP25 and TP26 on genomic DNA, we further investigated whether TP25 and TP26 had any effects on total RNA. For this purpose, different concentrations of TP25 and TP26 were incubated with total RNA of *M. luteus*. Nucleic acid electrophoresis shows that 150 μM of TP25 had a slight binding effect on RNA of *M. luteus*. When the concentration of TP25 increased to 300 μM, almost all total RNA of *M. luteus* was retarded. On the contrary, the control P86P15 had no effect on total RNA when its concentration was 450 μM (Figure 6B). Similar results were observed when TP26 was incubated with total RNA of *M. luteus* (Figure 6B). These findings suggest that TP25 and TP26 have a direct interaction with total bacterial RNA.

### Cytotoxicity effects of TP25 and TP26 on HT-29 cells

**Figure 7:**
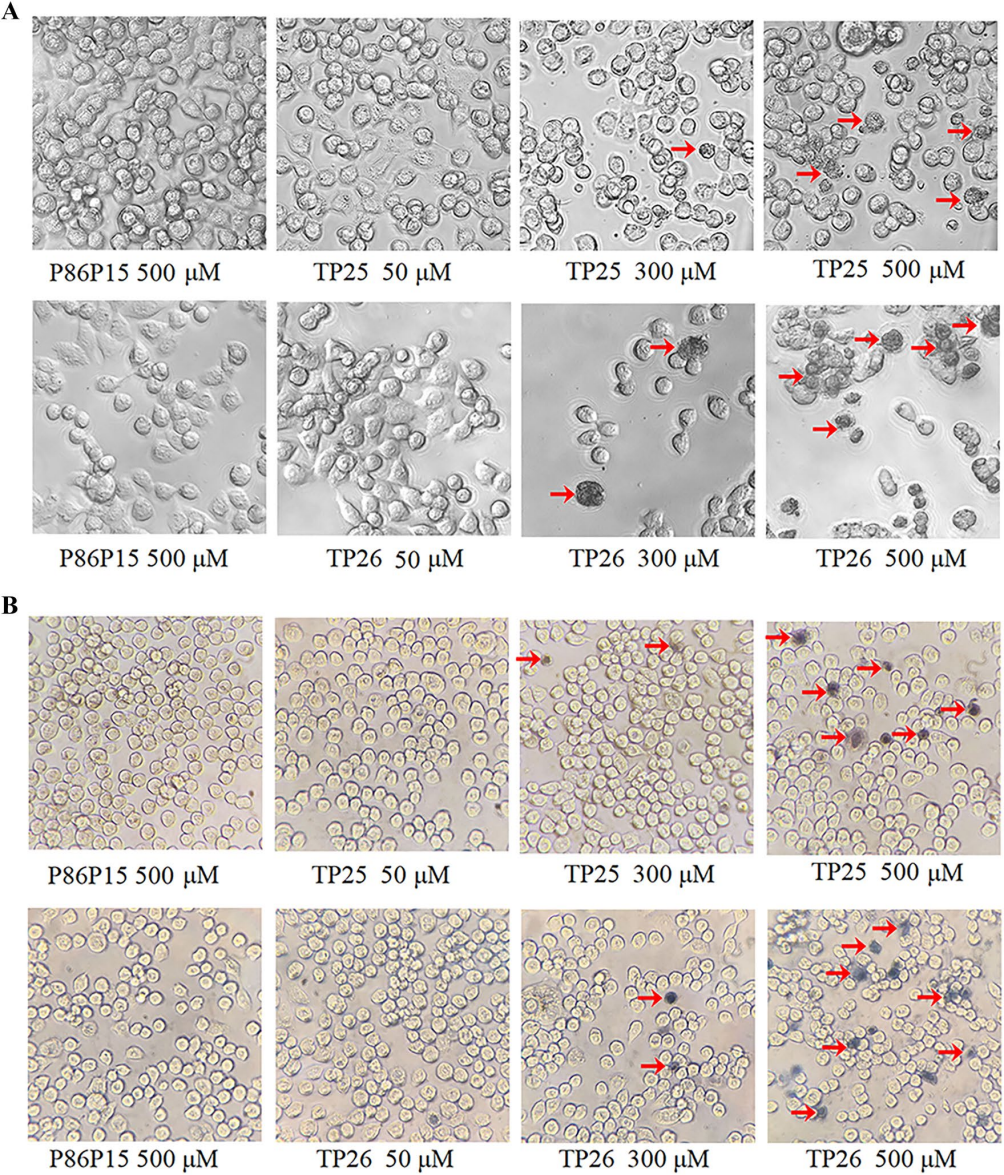
**Effect of TP25 and TP26 on the morphology of HT-29 cell lines.** HT-29 cells were treated with 50 μM, 300 μM or 500 μM of TP25 and TP26 for 24 h at 37 °C, then the cells were observed directly (**A**) or observed after dying with trypan blue (**B**) under the inverted phase contrast microscope. P86P15 was used as a control. **C** cytotoxicity of TP25 and TP26 on HT-29 cell lines evaluated by MTT assay. HT-29 cells were treated with different concentrations of TP25, TG 26, or P86P15 for 24 h at 37 °C. Then, the relative inhibition rate of HT-29 cells was determined by MTT assay. Values are shown as mean ± SEM (*N* = 3). N represents the number of times the experiment was performed. *P < 0.05; **P < 0.01.

Recently, anticancer activities of antimicrobial peptides have attracted more and more attention. In order to investigate the effects of TP25 and TP26 on human cancer cell lines, the HT-29 cell line was selected and the cytotoxic effects of TP25 and TP26 on it were evaluated. The results show that when TP25 and TP26 concentrations increased, the morphology of the cells changed gradually (Figures 7A, B). When the concentrations of TP25 and TP26 increased to 500 μM, a large number of necrotic cells appeared, which was confirmed by trypan blue assay (Figures 7A, B). In accordance with these results, the MTT assay shows that the relative inhibition rates of HT-29 induced by TP25 and TP26 were obviously enhanced as their concentration increased (Figure 7C). These results indicate that TP25 and TP26 have obvious cytotoxicity effects on HT-29 cells.

## Discussion

In this study, we examined the expression profiles of *PoTFPI-1* and *PoTFPI-2* in different tissues of flounder under normal physiological conditions and the result shows that *PoTFPI-1* and *PoTFPI-2* expression is distributed in all the tissues examined. The widespread tissue distribution may be one of its characteristics. For example, the wide tissue distributions of red drum TFPI-1 and TFPI-2 [28, 35], and of tongue sole TFPI-1 and TFPI-2 [29] were observed. Human TFPI-1 and TFPI-2 also displayed widespread tissue distribution. TFPI-1 was expressed by endothelial cells, monocytes, and macrophages [3], and TFPI-2 was synthesized and secreted by multiple cells, such as skin fibroblasts, endothelial cells, smooth muscle cells, dermal fibroblasts, keratinocytes, monocytes, and macrophages [13, 20, 36]. In addition, we found that high expression of *PoTFPI-1* and *PoTFPI-2* occurred in the liver, heart, blood, and intestines. High expression in the blood and heart were perhaps associated with the roles of TFPI-1 and TFPI-2 in blood coagulation mediated by a tissue factor pathway [22, 37], which was similar to the results found in the red drum [35].

Increasing evidence indicates that teleost TFPI is involved in antimicrobial immunity, and its expression has been considered to be induced by infection. For example, SoTFPI-1 and SoTFPI-2 expression in red drum kidneys were induced by both live *E. tarda* and LPS [28]. The expressions of *CsTFPI-1* and *CsTFPI-2* in tongue sole head kidney, liver, and spleen were enhanced upon infection by *V.* *anguillarum*, *S. agalactiae*, and ISKNV [29]*.* Similarly, in our study, *PoTFPI-1* and *PoTFPI-2* expressions in the head kidney, spleen, and liver were significantly induced by the extracellular bacterial pathogen *V. anguillarum*, the intracellular bacterial pathogen *E. tarda*, and the viral pathogen ISKNV. However, there were some differences between *PoTFPI-1* and *PoTFPI-2* expressions. The expression level of *PoTFPI-2* induced by three pathogens was basically higher than that of *PoTFPI-1*, which indicates their different role in antimicrobial immunity. It has been reported that TFPI originating from humans and teleosts displayed antimicrobial activity [28, 38], so it is likely that the induction of *PoTFPI-1* and *PoTFPI-2* is a general defense mechanism employed by the host during bacterial stimulation. In addition, considering that PoTFPI-1 and PoTFPI-2 are predicted to be secreted proteins localized in the extracellular space, we speculate that PoTFPI-1 and PoTFPI-2 may interact directly with pathogens, like recombinant human TFPI-1 that can bind with LPS [39].

Antibacterial activity of many peptides often depends on their net charge. One of the characteristics of the C-terminal of TFPI-1 and TFPI-2 is multiple positively charged amino acids, which display antibacterial potential. For example, C-terminal fragments of human TFPI-1 can kill serum-resistant *E. coli* through the complement pathway of the innate immune system [38]. Synthesized C-terminal peptides of human TFPI-1 and TFPI-2 possessed antimicrobial activity against Gram-negative and Gram-positive bacteria [40]. Recently, a number of vertebrate TFPI short peptides were synthesized and were used for antimicrobial assay. TC24 and TC38, originated from the C-terminus of CsTFPI-1 and CsTFPI-2; they possess antibacterial activity against *M. luteus* [29]. In red drum, the C-terminal peptide TO17 of TFPI-1 was synthesized and proved to be bactericidal against *E. tarda* [28]. C-terminal peptide TO24, derived from red drum TFPI-2, exerted its antibacterial activity by destroying cell membrane integrity, penetrating the cytoplasm and interacting with DNA and total RNA [41]. Similarly, in our study, we found that two synthesized peptides TP25 and TP26, based on the C-terminus residues of PoTFPI-1 and PoTFPI-2, possessed antibacterial activity against *M. luteus* and/or *S. aureus* and both TP25 and TP26 could penetrate into the cytoplasm and bind with genomic DNA and total RNA. These results imply that both TP25 and TP26 may exert their antibacterial effects through a mechanism different from that of human TFPI-1 and TFPI-2 fragments, and may be the same mechanism with that of red drum TO24 [41].

Recently, an increasing body of evidence has demonstrated that antimicrobial peptides are able to exert anticancer activities [42–44]. The defensins, isolated from different species, were among the first antimicrobial peptides to be discovered and to demonstrate antitumor activity [45]. Lactoferricin B shows antitumor properties by exerting lethal, selective destabilizing effects on cancer cell cytoplasmic and mitochondrial membranes [46]. As an ionophoric AMP isolated from the skin of the African clawed frog, Magainin II, acts as ion channels, leading cytolysis of cancerous cells [47]. In teleost, Zhou et al. reported that Pc-pis, a member of the Piscidin family from the large yellow croaker kills human cancerous cells such as HCT116, DU145 MCF7, and HeLa [48]. Likewise, in the current work, we found that TP25 and TP26 exhibited obvious cytotoxicity against HT-29 cancer cells. Although the mechanism behind this phenomenon should be further investigated, the results of the present study reveal that TP25 and TP26 are promising candidates to inhibit cancer cell growth.

In conclusion, we investigated for the first time the molecular features, tissue distribution, expression pattern of PoTFPI-1 and PoTFPI-2, and also detected the antibacterial and anticancer activities of two C-terminal peptides, TP25 and TP26, respectively. The results indicate that both PoTFPI-1 and PoTFPI-2 are expressed ubiquitously in multiple tissues. After stimulating with bacterial or viral pathogens, both *PoTFPI-1* and *PoTFPI-2* exhibited significant increased expressions in a manner that depended on the pathogen, tissue type, and infection stage. C-terminal peptides TP25 and TP26 displayed antibacterial activity and anti-cancer cell activities. These results imply the involvement of PoTFPI-1 and PoTFPI-2 in innate immunity and add new insight into the role of teleost TFPI.

## Data Availability

All data generated or analyzed during this study are included in this published article.

## Abbreviations

*E. coli*: *Escherichia coli*
*E. tarda*: *Edwardsiella tarda*
*K. pneumonia*: *Klebsiellar pneumonia*
*P. putida*: *Pseudomonas putida*
*M. luteus*: *Micrococcus luteus*
*S. marcescens*: *Serratia marcescens*
*S. agalactiae*: *Streptococcus agalactiae*
*S. aureus*: *Staphylococcus aureus*
*V. anguillarum*: *Vibrio anguillarum*
*V. harveyi*: *Vibrio harveyi*
*V. litoralis*: *Vibrio litoralis*
*V. parahaemolyticus*: *Vibrio parahaemolyticus*
*V. scophthalmi*: *Vibrio scophthalmi*
*V. vulnificus*: *Vibrio vulnificus*
SKNV: Infectious spleen and kidney necrosis virus
*P. olivaceus*: Paralichthys olivaceous
LB: Luria–Bertani broth
PBS: Phosphate-buffered saline
PCR: Polymerase Chain Reaction
RT-qPCR: Quantitative real-time reverse transcription-PCR
ORF: Open reading frames
NJ: Neighbor-joining
CFU: Colony-forming units
MIC: Minimum inhibitory concentration
MBC: Minimum bactericidal concentration
BHI: Brain Heart Infusion
